# Expected benefits and concerns regarding virtual reality in caring for terminally ill cancer patients – a qualitative interview study

**DOI:** 10.1186/s12904-024-01557-6

**Published:** 2024-11-04

**Authors:** Anja Greinacher, Bernd Alt-Epping, Christina Gerlach, Cornelia Wrzus

**Affiliations:** 1 Department of Palliative Medicine, Heidelberg University Hospital, Im Neuenheimer Feld 305, 69120 Heidelberg, Germany; 2 Institute of Medical Psychology, Heidelberg University Hospital, Bergheimer Straße 20, 69115 Heidelberg, Germany; 3 Clinical Psychology, Interaction- and Psychotherapy Research, Faculty of Social Sciences, University of Mannheim, L5,4, 68161 Mannheim, Germany; 4 Psychological Institute and Network Aging Research, Ruprecht Karls University of Heidelberg, Bergheimer Str. 20, 69115 Heidelberg, Germany

**Keywords:** Virtual reality, Expected benefits, Concerns, Palliative care, Oncology, Personalized therapy, Qualitative study

## Abstract

**Background:**

Many palliative cancer patients require inpatient hospital treatment for medical reasons, which contrasts their frequent desire to be at home. Virtual reality (VR) could be a way of bringing the home environment closer to them. First observations have shown benefits from VR for inpatients in palliative care. The aim of this qualitative, descriptive study was to explore the expectations of in-patients suffering from incurable cancer and their relatives about VR, in particular individualized VR images of the patients’ own home.

**Methods:**

Semi-structured interviews with inpatients suffering from incurable cancers and their relatives in three medical settings (palliative care, hematology, radiotherapy) of a German university hospital. Qualitative content analysis about expected benefits and concerns regarding VR-videos showing their private home; defining the main topics deductively and the subcategories inductively. We also assessed the patients’ subjective perspective on their remaining time to live to estimate the impact of double awareness on the results. The Patient Advisory Board informed the study protocol and conduct.

**Results:**

We interviewed 15 patients (8 men; age M = 63.4, SD = 11.34; range 39–82) under palliative care, and four relatives. We organized the interview content in 6 themes (general interest, desired content, non-desired content, expected benefits, concerns, and irregularities) and 26 sub-themes. Most patients and relatives were interested in using VR during hospital treatment. They often preferred viewing nature or tourist sites over seeing their home or family. Reasons could be linked to privacy concerns and the general desire for distraction from the current situation that they specified with their expectation of well-being, a break from the patient-experience, the pursue of curiosity, and the VR evoking fond memories.

**Conclusion:**

VR seems to be of interest for palliative cancer patients, especially as distraction and relief from their illness. The desired content can be very different, so a choice from a selection of VR-content should be made available. If patients want to see videos of their own home, recordings by relatives instead of study or hospital staff seem to meet the need for privacy.

**Trial registration:**

Registered at Deutsches Register Klinischer Studien; registration number: DRKS00032172; registration date: 11/07/2023. https://drks.de/search/de/trial/DRKS00032172.

**Supplementary Information:**

The online version contains supplementary material available at 10.1186/s12904-024-01557-6.

## Background

Many incurably affected cancer patients need hospital for treatment over long periods of time. This contrasts with the desire for a sense of normality in which they are not reduced to their patient role [1]. Further, a common phenomenon described in people living with incurable cancer is double awareness: the patient’s ‘capacity to simultaneously hold the idea of living and dying’ [2, 3], which may collude with the subjective future time perspective. Normality and shelter may be associated with home, so that most people with advanced cancer desire their own home to be their central place to be [4, 5]. Virtual reality (VR) could be a way to bridge the gap and bring the patient’s own home to the hospital bed.

VR is a computer technology that creates a three-dimensional image and thus imitates reality [6, 7]. Immersive processes create the feeling of being in VR [8]. Personalized videos can be created with the help of 360° recordings. VR has been used in health care since the 1980s [7, 8], e.g., to distract from visual and auditory stimuli that can cause anxiety, longing, and other negative feelings, and have a positive effect on symptom relief and well-being [9–13].

VR is regularly used in the treatment of cancer patients, because VR interventions may have positive effects during cancer treatment [14]. These positive effects include a reduced perception of pain, increased well-being, and more effective rehabilitation, also mediated by a reduced perception of pain during physical training. Well-being can be increased by reducing anxiety and depression using VR applications, creating positive emotions such as joy, and allowing users to relax or experience distraction from negative aspects of their condition.

In previous studies, patients either saw an identical video or could choose between several pre-recorded videos. Initial research on the potential of personalized VR in palliative care did not show additional benefits, which was attributed to the limited selection of VR video scenarios [15]. Two recent reviews showed that VR in a palliative context is useable, feasible, accepted by patients [16, 17], and has hardly any adverse effects [18]. So far, there is no research on individualized VR content, i.e. videos that are recorded according to the user’s wishes, although some studies demonstrated individualized images have a greater emotional effect on people [19, 20].

Thus, we explored the expectations of in-patients suffering from incurable cancer and their relatives about VR. We were particularly interested in expected benefits and concerns regarding individualized VR images of the patients’ own home.

## Methods

The interviews are part of a larger study on VR as an intervention for terminally ill cancer patients [21]. The report of this qualitative, descriptive study is guided by the Consolidated Criteria for Reporting Qualitative Research (COREQ) [22] to ensure methodological rigor.

### Aim and study design

We conducted semi-structured interviews with inpatients and family members to explore expected benefits and concerns about VR in the context of caring for terminally ill cancer patients. We were particularly interested in their opinions on 360° video recordings of their own home and family.

### Setting

From June 6th, 2023 to October 11th, 2023, the interviews took place on three wards at the Heidelberg University Hospital that care of palliative cancer patients: palliative care, hematology, and radiotherapy. The patients were interviewed in their hospital rooms, sometimes with their room neighbours present. The Heidelberg University Hospital is a German tertiary care center delivering comprehensive cancer care in the participating wards for 1200 patients annually.

### Participants

The inclusion criteria were: age 18 years or older; diagnosis of an incurable oncological or haematological disease (or malignant haematological disease with uncertain prognosis), or being a family member of such a patient; and capability to consent. Patients were excluded when they were in a poor general condition, showed cognitive or communication deficits, or had only a few days of life expectancy as assessed by the physicians in charge for the patients on the wards. Hospital physicians identified patients that could be asked for participation based on their professional impression of the physical and mental state of patients. As both physical and mental states could vary from day to day, no systematic screening was used as that would have been too demanding for both patients and physicians. Relatives were excluded when they felt too burdened to participate, had cognitive or communication deficits, or the patient was close to death. All patients that met the inclusion criteria during the study period were asked to participate in the study. To control selection bias, we monitored the admission count on the palliative care and the haematology ward.

### Data collection

After identification of eligible patients by the attending physicians, study staff approached the patients and their relatives, informed about the study, and invited them to the interviews. After informed consent, we interviewed the participants; we gave space and respect to further topics and thoughts that deviated from the study questions to respond to possible emotional distress. Afterwards, participants were asked for demographic information and length of hospitalization (patients). The interviews were audio-recorded on tablets (Xiaomi Pad 5, 11 Zoll), which were also used to enter demographic information in a digital questionnaire. Two research assistants (f, graduating in psychology) conducted the interviews in person between June and October 2023. Both research assistants possessed substantial communication skills learned during the BSc and MSc studies in psychology as well as clinical internships. Additionally, they were trained for the interview protocol and supervised by CW (f, psychology professor). Study participants did not know the study staff beforehand. Before the interview, they introduced themselves, explained the study, and made another appointment for the interview if required. During the analysis of the interviews, it became clear that the topic of privacy regarding VR videos of the own home was very important. For this reason, CG (f, MD, palliative care physician, oncologist) conducted two further interviews in October 2023 to explore the topic of privacy in greater depth.

### Semi-structured interviews

After a thorough literature search, CW developed a semi-structured interview guide [23] (see supplementary material 1), which was discussed with CG and BAE (m, professor, palliative care physician, and oncologist). Before the interviews, we sought advice from the Patient Advisory Board of the National Center for Tumor Diseases Heidelberg (NCT Heidelberg) on the interview guide [21].

After a brief introduction and explanation of VR, we asked about five topics: (I) general interest in VR, (II) potential content patients would be interested in, (III) expected benefits, (IV) concerns, and (V) time perspective (supplementary material 1). While we asked all patients the main questions, we adapted the clarifying follow-up questions to what each participant had said. Further, we supplemented the exploration of the desired content by asking the patients and relatives to weigh their choices against other content with the help of a visual analogous scale (VAS), how much they would like to see a particular content (home, family and friends, cites and cities, nature) from 0 (not at all) to 10 (absolutely). In the end, we asked about the patients’ agreement on the feeling of having time left and the feeling that time is running out with the help of a VAS.

### Data analysis

The recorded interviews had a mean length of 10.29 min (*M* = 4.19 min; range: 3.57–20.11 min) and were transcribed verbatim. Due to the significant medical conditions of the patients, the transcripts were not returned to them for validation. They were analyzed by the author AG (f, postdoctoral psychologist, psychotherapist) and a research assistant. We used the computer-assisted qualitative data analysis software MAXQDA 2022 (VERBI Software, 2021) to organize the content analysis [24]. We defined the main topics of the analysis deductively according to the topics in the semi-structured interviews, i.e. theory guided. The topics set the category system’s base of the analysis. The subcategories were developed inductively, i.e. from the interview material (Mayring, 2010). With MAXQDA, we labelled semantic units, consisting of words, sentences or paragraphs with codes; and grouped the codes into more abstract (sub-)categories. Individual codes could be assigned to several (sub-)categories. After the analysis of 12 interviews (70.6%), AG and a research assistant discussed the category system critically, adjusted it, and reassigned the codes if necessary. The research team discussed inconsistencies between coders on weekly meetings until consensus was reached, and new aspects were integrated in the iterative analysis. We analyzed sociodemographic variables as well as the answers on the visual analogous scale with descriptive quantitative methods (frequency distribution, mean scores, standard deviation, and range).

## Results

**Fig. 1:**
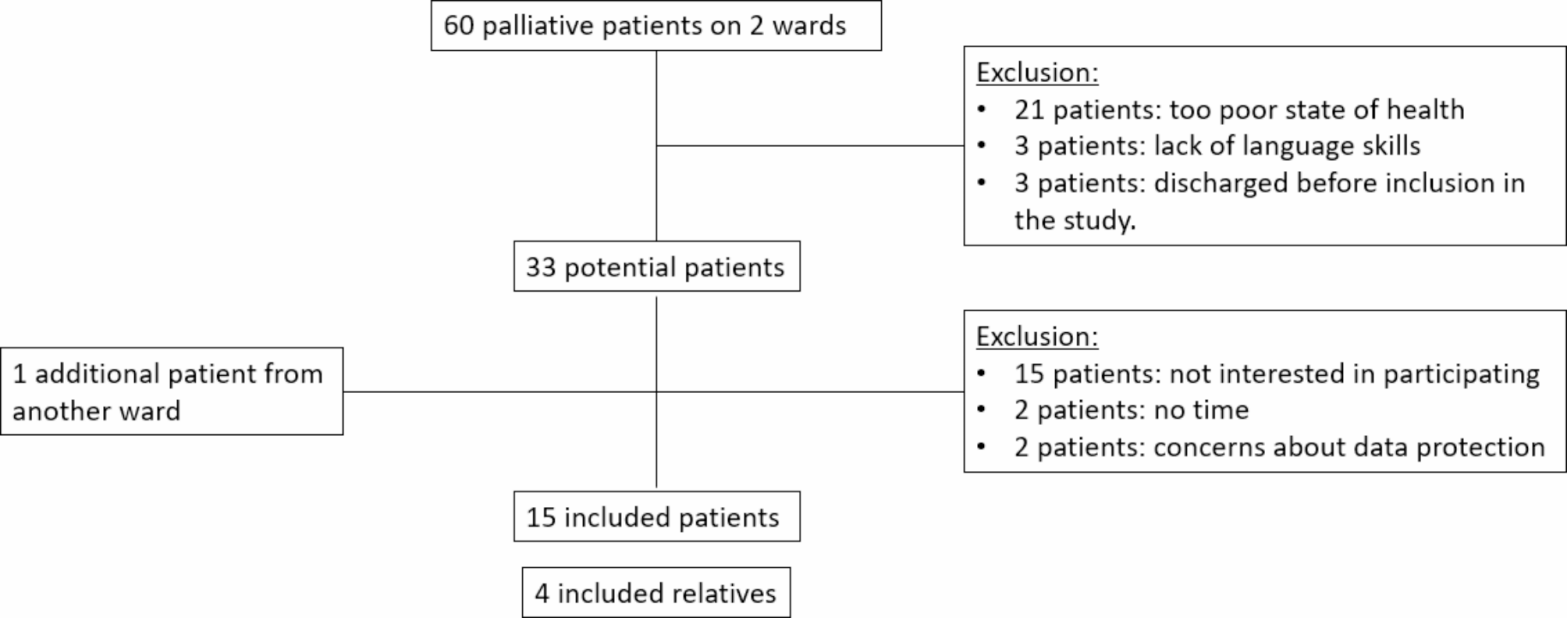
Flowchart of included study participants

During the interview period (5 months), from the two units monitored for selection bias a total of 60 palliative patients were admitted, of which 33 patients met the inclusion criteria. From these 33 potential participants, 15 patients were not interested to participate, two patients had no time, and two patients indicated concerns with data protection (Fig. 1). The 15 participants, 8 men and 7 women, ranged in age between 39 and 82 years (*M* = 63.4; *SD* = 11.3). The majority was married (*n* = 8) or single (*n* = 7), two were widowed, one did not provide information. Six patients had children. The majority were diagnosed with multiple myeloma (*n* = 5), *n* = 3 patients had a pancreatic carcinoma while the following diagnoses occurred once: gastric cancer, cholangiocellular carcinoma, colorectal carcinoma, breast cancer, ovarian cancer, and lymphoma. On average, patients had been in the hospital for 8.9 days (SD = 10.7). We did not collect the socio-demographic details of the relatives.

We identified a total of 235 codes and classified them into 6 main categories with subcategories: General interest in VR, desired content, content not desired, expected benefits, concerns, irregularities (see Table 1). Of the 235 codes, 42 were assigned to two different subcategories. 

**Table 1:** Category system with main and subcategories

	*n*_codes_	%	*n*_participants_	%	*n*_relatives_		%
General interest in VR							
Interest	15	6.28	9	60.00	3		20.00
Lack of interest	7	2.98	5	33.33	0		
Undecided	1	0.43	1	6.67	0		
Desired VR content							
Nature	25	10.64	10	66.67	2		13.33
Cities and sights	8	3.40	5	33.33	1		6.67
Home and family	3	1.28	2	13.33	1		6.67
Escape from hospital	3	1.28	3	6.67	0		
Time travel	2	0.85	2	13.33	0		
Games	1	0.43	1	6.67	0		
Non-desired VR content							
Boring content	6	2.55	5	33.33	1		6.67
Negative images	6	2.55	5	33.33	0		
Expected benefits							
Well-being	22	9.36	14	93.33	2		13.33
Break from patient experience	9	3.83	5	33.33	1		6.67
Evoke fond memories	8	3.40	3	6.67	2		13.33
Pursue curiosity	4	1.70	2	13.33	1		6.67
Uncertain	4	1.70	3	6.67	1		6.67
Concerns							
No concerns	11	4.68	7	46.67	3		20.00
Privacy and security							
Conditions	10	4.26	4	26.67	2		13.33
Unimaginable	7	2.98	4	26.67	1		6.67
No concerns	7	2.98	5	33.33	1		6.67
Problems with implementation	18	7.66	10	66.67	3		20.00
Concerns of those filmed	14	5.96	11	73.33	2		13.33
No added benefits	7	2.98	6	40.00	0		
Negative feelings/memories	7	2.98	5	33.33	1		6.67
Influence through use of VR	5	2.13	3	6.67	0		
Irregularities							
Thoughts about others	8	3.40	5	33.33	0		
Contradictions	7	2.98	4	26.67	0		
Question not understood/answer unsuitable	10	4.26	6	40.00	0		

*Note* Results from the content analysis. Deductive, theory-guided main categories and inductive subcategories of the content analysis with number of assigned codes and number of participants who mentioned the categories. Only patients were asked about their time perspective. n_codes_ = number of codes assigned to subcategories; n_participants_ = number of participants who mentioned codes assigned to subcategories; n_relatives_ = number of relatives who mentioned codes assigned to subcategories

### General interest in VR

Most patients and relatives expressed a general interest in VR for cancer patients in the palliative setting. For example, one relative said: *“And I would definitely be in favor of something like that. Absolutely” (rel04f).* Some patients reported a clear lack of interest: *“Because I’m not interested in it*,* I don’t deal with it at all” (pat12f).* One patient remained undecided: *“I can’t imagine at the moment because I always prefer the original. But maybe in old age or*,* never say never*,* with advanced illness*,* but I’ve already mentioned it*,* I’m actually gifted with a great imagination” (pat13m).* Patients and relatives named content that they would like to see for VR applications in the palliative setting.

### Desired VR content

Most patients and half of the relatives named nature as desirable content for the VR videos. Very different aspects were mentioned, including general videos with *“everything to do with mountains” (rel04f)*; walks in the forest or *“by the sea*,* on the beach” (pat01m)*. But there were also specific places mentioned such as *“my meadow” (pat09m)*. Cities and sights were also frequently named by patients and relatives. Here, too, specific places such as *“Jerusalem” (rel03m)* were mentioned as well as general ideas: *“…or simply pretty*,* great cities*,* pretty buildings…” (pat09m)*. Some patients and one relative actively named their own home and family as videos that they would like to see, e.g. *“… therefore our home. We built the house ourselves” (rel01m).* For some patients, the content was less important, but they were interested in a virtual escape from the hospital. Time travel to the future or the past was also of interest. One patient would be interested in playing games on VR headsets.

### Weighing of interest

Nature (*M* = 7.53) and cities and sights (*M* = 8.18) were endorsed most strongly; the own home (e.g. living room, garden; *M* = 5.00) and images of people close to them (e.g. friends, family; *M* = 7.00) were moderately preferred with substantial differences between people (Table 2, column range).

**Table 2:** Scaled retrieval of desired video content

	*n*_participants_	%	*n*_relatives_	%	M	range
Nature	11	73.33	3	75.00	7.53	0–10
Cities and sights	11	73.33	3	75.00	8.18	5.5–10
Home	11	73.33	3	75.00	5.00	0–10
Family, friends	11	73.33	3	75.00	7.00	0–10

*Note* Patients and relatives were asked directly which content they would like to see on a scale from 0 (not at all) to 10 (absolutely). Only participants were asked who stated general interest in VR

### Non-desired VR content

In addition to the desired videos, the study participants named content that they would not want to see on VR headsets. Some negative images such as terror, war, torture, and disputes were named. However, boring content were also highlighted; one patient was concerned that *“some games are so optimized for the disease or for tests running in the background that they are simply boring” (pat09m)*. Nevertheless, for patients and relatives, the idea of seeing a personalized VR video was clearly associated with benefits.

### Expected benefits

Around half of the interviewees anticipated well-being such as *“joy” (pat04f)*, *“good well-being*,* positive experiences” (rel03m)*, and fun. They expected that the videos would evoke fond memories. One relative said: *“… when you can visually go back to these places that you actually love. […] and you also know what you experienced there*,* out in the garden. It brings back a lot of memories…” (rel01m)*. The interviewees also expected a break from the patient experience. Some saw an advantage in being able to pursue their own curiosity. A few patients and one relative had little idea of the positive effects a VR video could have in a palliative context and were uncertain. One patient said: *“That’s a difficult question. If I don’t try it now*,* I can’t do it now*,* so I can’t give you an answer to that. I’m sorry” (pat07m).* Next to the expected benefits, we were interested in concerns.

### Concerns

When asked about concerns, most patients and relatives spontaneously replied that they had no concerns. The interviewer raised possible concerns, some of which were then shared by the interviewees. Privacy and security were frequently discussed in the context of videos of the patient’s own home. Some patients and one relative did not perceive it as a problem at all. However, the majority of the participants mentioned conditions that would have to be met; usually who would be allowed to record the videos and who would be allowed to see them, e.g. *“…as long as they [note: individualized videos] are only accessible to these specific people*,* that wouldn’t be a problem for me at all.” (rel04f)* or *“So I would only want my son to do that [note: video recording of my own home]” (rel04f).* Most patients also considered the fact that relatives would be filmed to be unproblematic. However, some interviewees considered personalized VR videos of their own home and/or relatives unimaginable due to privacy concerns.

Most patients and relatives had no concerns specifically about the implementation of personalized videos to patients in the palliative setting. If they mentioned concerns, they mostly asked for support, e.g. “…*if you are no longer quite up to scratch motorically… you have to make sure that you put it on well*,* that it hasn’t slipped somehow*,* otherwise you see everything askew and that’s not good either. Or if you don’t put it on properly*,* you can still see something from normal reality. It has to be put on properly*,* the straps have to be tightened; you might need a bit of help” (rel03m).* Some patients mentioned concern about not getting any added benefits from an individualized VR video. One patient said: *“I just thought to myself*,* yes*,* it’s virtual*,* but not real. And that can make her happy at the moment*,* but when she’s out of the thing again*,* just shitty again*,* right?” (pat14f).* Others were concerned that individualized videos would reinforce negative feelings or memories, including homesickness. One patient said: *“…in places that you have a close connection to. […] I can’t really tell*,* but I think it’s quite possible that it triggers sadness. That it’s a pain*,* a shock*,* an internal shock*,* along the lines of: ‘Look there. You’ll never be able to do it again.’ […] or look at the tree you planted on your x-th birthday and how big it is. But you won’t live to see it stand for 25 years because you have a lethal tumor. Although it doesn’t necessarily have to be deadly or won’t be deadly with treatment. But I’m afraid those negative thoughts will come” (pat09m).*

Some patients were concerned about being influenced through the use of VR, e.g. that VR videos *“lead to an impairment of the own fantasy world” (pat14f)* or that they *“actually lose my sense of reality” (pat14f)* as a result. One patient mentioned the fear of being manipulated by VR videos and drew a reference to marketing strategies.

### Time perspective

Patients agreed moderately (*M* = 4.83) with the statement that they still have time to make plans. They also agreed moderately (*M* = 5.40) that their time is running out. The answers to both statements showed substantial differences between the patients (see Table 3, column range).

**Table 3:** Time perspective of patients

	*n*_participants_	%	M	range
“I still have a lot of time in my life to make new plans.”	15	100.00	4.83	0–10
“I have the feeling that my time is running out.”	15	100.00	5.40	0–10

*Note* Patients were asked about their time perspective on a scale from 0 (not at all) to 10 (absolutely)

### Irregularities

In 10 answers (by 6 patients), we think that the interviewee had not understood the question correctly, as the answer was unsuitable to the question, which might be due to the vulnerable health of the patients. Further, we observed in seven statements that the participants contradicted themselves or changed their mind during the interview, which might occur in any reflective context. It was also noticeable that thoughts about others were stated, such as *“There are certainly people and situations for whom that would be nice. But as I said*,* I prefer to look at it globally somehow” (pat14f).* Or *“Oh well*,* not for me personally*,* I don’t have a garden*,* but I think it would be really interesting for older people” (pat05m)* that could be attributed to double awareness in order to cope with being under palliative care, facing a life limiting prognosis.

## Discussion

We explored expected benefits and concerns of VR in 15 inpatients suffering from incurable cancer, and their relatives. Most participants and relatives showed a general interest in VR applications. Sometimes, participants expressed the concern that they did not see any advantage in the VR application. Less frequently, it was assumed that VR could have a negative impact. Most participants hoped that the videos would provide them with positive emotions, distraction from their experience as patients, and the activation of fond memories. In this context, we tested the assumption that their home is the preferred place for most patients at the end-t of-life. Previous research indicated that many people are strongly attached to their home, neighbourhood, and region, and often consider their home a part of themselves [25, 26]. However, other than expected from theory that patients would prioritize to experience their home environment, the palliative cancer patients and their relatives named nature shots and tourist destinations as particularly interesting for VR content. One reason may be, that the participants were concerned about privacy and security violation - especially in the context of videos of their own homes. A second reason may be that patients did not feel to be at their end-of-life or that they nevertheless preferred to make plans, in a sense of double-awareness, because we found a wide variety in their subjective future time perspective.

VR has been used in health care increasingly in the past decades, but usually not in a palliative context [7]. Nowadays, the availability and easy usage of VR headsets enable a widespread implementation of VR applications, yet VR should only be implemented after considering patients’ and relatives’ opinions, desires, and concerns. Most of the patients and relatives we interviewed considered VR applications to be a good option for people suffering from incurable cancer. This result is in line with two recent systematic reviews [16, 18] (one with a meta-analysis [16]), and a scoping review [17], which classify VR as usable, feasible, and acceptable in a palliative context. However, the reviews criticized the lack of evidence for the effectiveness of VR in the palliative setting. So far, VR content from other settings was transferred to the situation of patients under palliative care. Only one Japanese study [27] tested VR content in 20 inpatients in palliative care addressing a travel aim of the patient’s choice and observed more pronounced effects with VR content that was associated with personal memories. Patients in our interviews also wished to travel virtually both to places on their ‘bucket list’ and to places associated with fond memories. Noteworthy, the Japanese study was neither designed to explore patient wishes nor to differentiate between memorable vs. new travel aims. The feasibility and effects of virtual visits to places or settings chosen by the patients on a personalized base was recently explored [15, 28]. Yet, these studies depended on the availability of respective VR content, and did not consider the patients’ own home.

Seeing one’s home or family in the VR videos was not as desired as we anticipated based on the literature and clinical experience that palliative patients prefer to be at home. Four reasons for this emerged from the interviews: First, patients reported that their relatives come to visit them in hospital often enough, making VR content of the home environment dispensable. Second, patients would rather use the VR glasses for something unknown, e.g., places they have not been to before. This result is in line with the anticipated benefits of experiencing distraction and increased well-being through VR experiences [27, 29]. However, it also contrasts with previous findings on future time perspective that people with a limited time perspective prefer to experience familiar situations [30]. This might be explained by the fact that new experiences with the help of VR require little investment. Third, the heterogeneity of the subjective future time perspective illustrated that some patients may block out their end-of-life, simultaneously wishing home to be their place of death. Fourth, the patients and relatives described some privacy concern. In particular, the idea of strangers coming into the home to record images for the VR video was rejected. These concerns should be taken very seriously: On one hand, they could prevent patients from foregoing potentially helpful VR videos of their own home. On the other hand, it could have negative effects if the videos are associated with a feeling of invasion of privacy. After these concerns became clear, we developed the idea that relatives could create the videos themselves with the help of consumer action cameras. This solution seemed conceivable for patients and relatives.

Our results reflect the points Pittara et al. identified [14] as important factors in the development of VR applications for palliative patients: In addition to demographic data and medical history, patients’ interests and everyday activities should be considered for VR experiences. In this context, the dignity and autonomy of patients need to be taken into account seriously [31]. This approach favours a very individual planning of VR applications for patients in palliative care.

### Strength and limitations

Making the patients’ voices heard is one of the strengths of this study. Although it is a monocentric study, we achieved to include participants from diverse units with integrated palliative care (specialist palliative care, haematology, radiotherapy), and heavily involved the patient advisory board. Nevertheless, the results of the study should not be generalized to all patient groups. It should also be kept in mind that there could be a selection bias when agreeing to the interviews about VR in palliative care and thus the majority of patients who participated were generally interested in VR. For the first time, the patient’s home was explored for VR content. Further, we integrated for the first time future time perspective to estimate the impact of double awareness on the participants’ expected benefits and concerns regarding an intervention for patients at the end-of-life.

### Future research

Following this interview study, an intervention study should examine whether patients in palliative care indeed benefit from individualized VR videos of their home. Privacy concerns should be considered and relatives could be asked to carry out the recording. Further studies should include people with illnesses other than cancer to gain a better understanding of the needs of different patient groups. It should be tested whether individualized videos have a more positive effect than pre-recorded videos from which patients can choose. In all intervention studies, attention should be paid to the fact that (individualized) VR videos could also have a negative effect, such as homesickness. One solution could be to always let the patients themselves decide what content they want to see.

### Conclusion and research implications

More than half of the palliative cancer patients and all relatives in our sample were interested in viewing VR. It became clear that patients should have the possibility to choose the content for their VR application, e.g., from a selection of nature and tourist sites, but also their own home and family. The latter was not as desired as expected from theory. Privacy concerns might be one aspect that can be addressed pragmatically through providing consumer action cameras to relatives, who then record the videos themselves.

## Electronic supplementary material

Below is the link to the electronic supplementary material.

Supplementary Material 1

## Data Availability

The data cannot be shared publicly because the interview transcripts contain personal data, including information that may allow personal identification of participants. Request for access to data can be addressed to Dr. Christina Gerlach (christina.gerlach@med.uni-heidelberg.de).

## Abbreviations

f: Female
m: Male
NCT Heidelberg: National Center for Tumor Diseases Heidelberg
Pat: Patient
VR: Virtual Reality
