# Supplementary material for: Expected benefits and concerns regarding virtual reality in caring for terminally ill cancer patients – a qualitative interview study
Source: BMC Palliat Care. 2024 Nov 4;23:254. doi: 10.1186/s12904-024-01557-6 (PMC11533371; doi:10.1186/s12904-024-01557-6)
Supplement: Supplementary file 1 — Supplementary Material 1 [file 12904_2024_1557_MOESM1_ESM.docx]

Interview guideline that was developed for the study.

| **I General interest:** I explained that you can see all sorts of different places and things with VR glasses; a walk in the forest, the Eiffel Tower, different cities around the world. Would that be something for you? Would you be interested in watching such videos with VR glasses? | | |
| --- | --- | --- |
| *➔ YES:* | |  |
|  | **II Content:** |  |
|  | Main questions | Follow-up questions |
|  | Which places would you expect to have a positive effect on your state of health if you could see them? | a) Are there any places you have enjoyed visiting and would like to see again?  b) Are there any places you would like to see but haven't yet?  c) Would you be interested in seeing recordings of your home?  d) Would pictures of people close to you be interesting for you?  e) What would you not like to see? |
|  | I have various categories here and would like to ask you which you would find helpful. Can you please tell me a number from 0 - „not at all“ to 10 – „absolutely“. | *(Name categories one by one and show printed scale: 0 – „not at all“; 5 – „neutral/undecided“; 10 – „absolutely“)*  a) Nature shots  b) Cities and sights  c) Your home, e.g. living room, garden  d) Recordings of people close to you, friends, family |
|  | **III Expected benefits:** |  |
|  | What would you hope to gain from being able to view such recordings? | What improvements or changes would you like to see or hope for? |
| *➔ IF NO and otherwise after possible benefits:* | | |
|  | **IV Concerns:** |  |
|  | In your opinion, what are the reasons not to watch such videos? What concerns do you have? | a) What concerns might you have about the technical implementation?  b) If you would like to experience your home with virtual reality, we would also have to make recordings there. This is, of course, a matter of your privacy and the video would only be available to the person for whom it was created. Under what circumstances would that be acceptable to you or do you reject such recordings in principle?  c) May I ask you another question about your concerns regarding recordings of your home? Can you describe what makes the difference for you between videos of desired destinations (examples if applicable) and your own home?  d) If we were to film friends and relatives for you for fifteen minutes, what concerns would you or possibly your friends and relatives have?  e) Recording videos for VR headsets is technically quite simple these days. What do you think if, for example, relatives or friends were to make these recordings instead of “strangers”? |
|  | **V Time perspective:** | |
|  | “I still have a lot of time in my life to make new plans.”  “I have the feeling that my time is running out.” | *(Show printed scale: 0 – „not at all“; 5 – „neutral/undecided“; 10 – „absolutely“)*  Please indicate your agreement with these two statements on a scale from 0 to 10. |

Note. After a brief introduction of the interviewer and the research project, the interview was conducted. The main questions were asked to all participants; the follow-up questions depending on the answer.
